# A floppy infant without lingual frenulum and kyphoscoliosis: Ehlers Danlos syndrome case report

**DOI:** 10.1186/s13052-021-00984-y

**Published:** 2021-02-12

**Authors:** Rosaura Conti, Chiara Zanchi, Egidio Barbi

**Affiliations:** 1 Department of Medicine, Surgery, and Health Sciences, University of Trieste, Trieste, Italy; 2 Institute for Maternal and Child Health, IRCCS “Burlo Garofolo”, Trieste, Italy

**Keywords:** Ehlers-Danlos, Floppy, Kyphoscoliotic, Frenulum, Case report

## Abstract

**Background:**

Ehlers-Danlos syndrome (EDS) represents a group of connective tissue disorders characterized by the fragility of the soft connective tissues resulting in widespread skin, ligament, joint, blood vessel and internal organ involvement. The clinical spectrum is highly variable in terms of clinical features, complications, severity, biochemical characteristics and genes mutations.

The kyphoscoliotic type EDS (EDS VIA) is a rare variant of the disease, with an incidence of 1:100.000 live births. EDS VIA presents at birth as severe muscular hypotonia, early onset of progressive kyphoscoliosis, marked hyperelasticity and fragility of the skin with abnormal scarring, severe joint hypermobility, luxations and osteopenia without a tendency to fractures. This condition is due to a mutation in the PLOD1 gene, and less commonly in FKBP14 gene, which results in the erroneous development of collagen molecules with consequent mechanical instability of the affected tissue.

**Case presentation:**

A female newborn, found to be floppy at birth, presented a remarkable physical examination for joint hypermobility, muscle weakness, hyperelastic skin, a slight curve of the spine, the absence of the inferior labial and lingual frenulum. Due to severe hypotonia, neuromuscular disorders such as Spinal Muscular Atrophy (SMA), genetic diseases such as Prader Willi syndrome (PWS), myopathies and connective tissue disorders were considered in the differential diagnosis. Targeted gene sequencing were performed for SMN1, PLOD1, FKBP14, COL6A1, COL6A2, COL6A3. The urinary lysyl and hydroxy-lysyl pyridinoline ratio was diagnostic before discovering the homozygous duplication in the PLOD1 gene, which confirmed kyphoscoliotic EDS diagnosis.

**Conclusion:**

In front of a floppy infant, a large variety of disorders should be considered, including some connective diseases. The presence at the birth of kyphoscoliosis, associated with joint hypermobility and the absence of the lingual and lower lip frenulum, should suggest an EDS.

## Background

Ehlers- Danlos syndrome represents a group of connective tissue disorders, which vary in terms of clinical features, biochemical characteristics and gene mutations.

The hypermobile type and the classical type represent more than 90% of all cases [1].

The kyphoscoliotic type of Ehlers-Danlos syndrome (EDS VIA) is a rare autosomal recessive disorder with an incidence of 1:100.000 live births, which occurs in the early years of life and mainly affects the musculoskeletal system [2].

It is caused by the homozygous mutation in PLOD1 gene, which codes for a collagen-modifying enzyme called lysyl hydroxylase 1 (LH1) [3]. Over 20 different mutations have been reported in PLOD1, the most common of which is a duplication of exons 10–16, which represent approximately 25% of all reported mutations. However, no relationship between position, type of mutation in the PLOD1 gene, and the severity of the clinical phenotype were observed so far [4]. Lysyl hydroxylase 1 (LH1) deficiency results in under-hydroxylation of collagen lysyl residues and, hence, impaired crosslink formation with consequent mechanical instability of the affected tissue [5].

The enzyme deficiency gives rise to the abnormal pattern of Lysyl Pyridoline (LP) and Hydroxylysyl Pyridoline cross-links (HP) formed in vivo and excreted in the urine. The ratio of total urinary LP to HP (LH/HP ratio) in patients with EDS VI is high compared with normal controls, and it is diagnostic for the disease [5].

Less commonly, it can be caused by a mutant form of FKBP14, which results in the erroneous development of cross-links between collagen molecules too, but in this case, the LH/HP ratio is normal [6].

EDS VIA is characterized, at birth, by severe muscular hypotonia and progressive and early onset kyphoscoliosis, marked skin hyperelasticity, fragility with abnormal scars, severe joint hypermobility, luxations and osteopenia without a tendency to fractures. Sometimes there is a Marfanoid habitus, a microcornea and a bluish sclera; occasionally, the rupture of arteries and eye globe is described. Intellect is usually unaffected [1, 2]. The diagnosis is guided by three major clinical criteria and ten minor clinical criteria, but confirmatory molecular testing is obligatory to reach a final diagnosis.

Long term outcomes in patients with EDS VIA, concern the possibility of walking, because of the hip dislocations, described in 25% of patients at birth, the restrictive lung disease due to the severe kyphoscoliosis and the vascular ruptures which represent the major life-threatening complication in this disorder [2].

## Case presentation

A female newborn appeared as a floppy infant at birth. She was born at term after an uneventful pregnancy, in which no abnormal fetal movements were detected. She was the only child of healthy consanguineous parents of Macedonian origins and her family history was not relevant.

Auxologic values for weight, length and head circumference were 3060 g (10°-50° p), 51,5 cm (50°-90° p) and 34 cm (10°-50° p), respectively. Apgar score was 9–10.

**Fig. 1:**
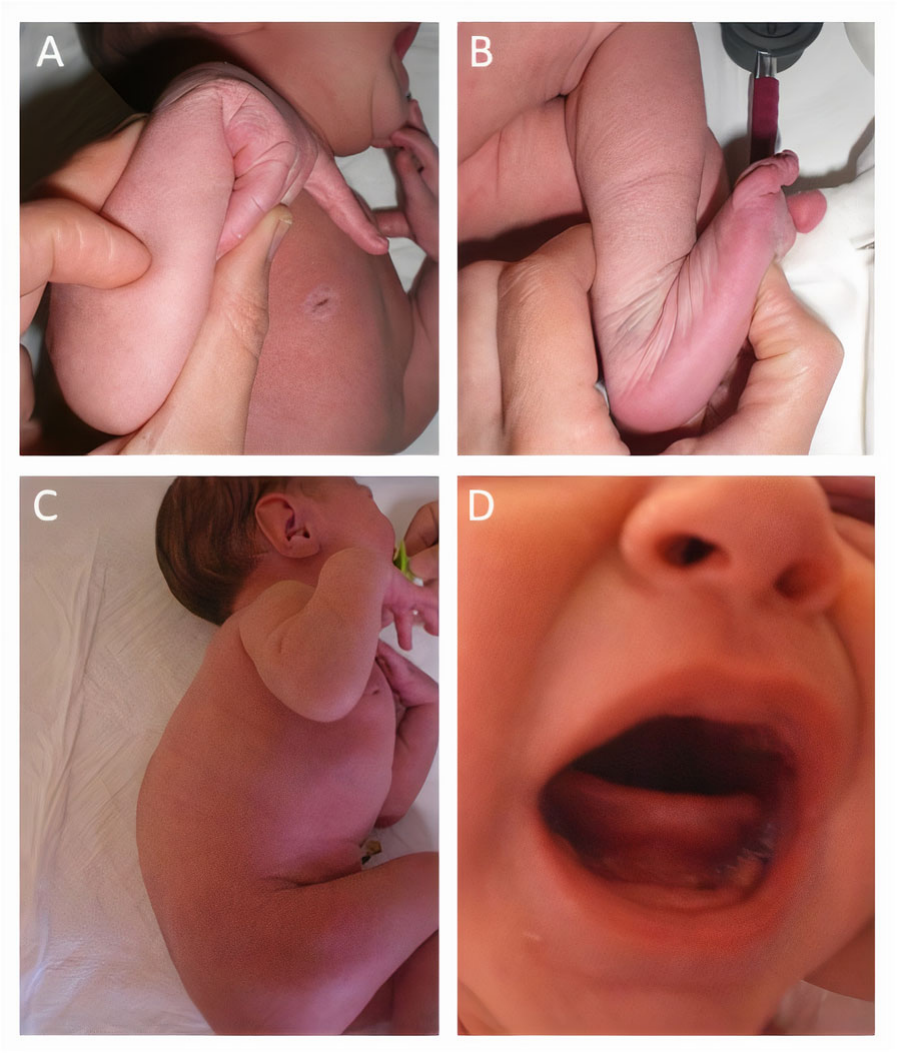
Neonatal presentation of Ehlers Danlos Syndrome kyphoscoliotic type. **a** and **b** joint hypermobility. **c** Early-onset kyphoscoliosis. **d** absence of inferior labial and lingual frenulum

Physical examination was remarkable for joint hypermobility, characterized by greater than usual wrist and ankle bending without affixing resistance during passive mobilization, hyperelastic skin, muscle weakness, poor antigravity movements on stimulation of the limbs, “frog” leg posture, a slight curve of the spine, and the absence of the inferior labial and lingual frenulum (Fig. 1).

Hypotonia, congenital or early-onset kyphoscoliosis, and generalized joint hypermobility are primary clinical criteria that allow, if coexisting, to perform the EDS VIA’s clinical diagnosis.

The normal extended metabolic screening ruled out organic acidemias, fatty acid oxidation disorders, amino-acid disorders and lysosomal storage disease. Normal creatine kinase (CK) levels, blood gas analysis and lactate were not supportive for a diagnose of dystrophies, congenital or mithocondrial myopathies. The brain magnetic resonance was normal as well. Genetic testing was performed firstly with DNA methylation analysis to exclude a PWS, then with targeted gene sequencing, for SMN1 to rule out a SMA. Normal PLOD1 and FKBP14 apparently excluded an EDS, while negative testing for COL6A1, COL6A2 and COL6A3 ruled out a Bethlem myopathy and a Ullrich dystrophy, that could both present with near normal values of CK.

The measurement of the urinary lysyl and hydroxy-lysyl-pyridinoline ratio tested positive for an abnormal pattern of lysyl pyridinoline and hydroxy-lysyl-pyridinoline crosslinks with a high ratio of deoxypyridinoline and pyridinoline crosslinks in the urine.

For this reason, even if targeted sequencing tested negative for mutations in PLOD1 and FKBP14 gene sequence, further genetic study through Multiple Ligation dependent Probe Amplification (MLPA) was performed. A homozygous duplication of exons 10–16, c1067_1846dup, in the PLOD1 gene, on chromosome 1p36.3-p36.2 was discovered, confirming the diagnosis of Ehlers-Danlos type VI A syndrome.

## Discussion and conclusion

In front of a floppy infant, a large variety of disorders must be considered, ranging from genetic and chromosomal anomalies (e.g., Down Syndrome, Prader Willi,), neuromuscular diseases, peripheral neuropathies, inborn errors of metabolism, infantile botulism, SMA, congenital muscular myopathies and connective disorders [7, 8]. A complete neuromuscular workup is usually recommended, but accurate physical examination can orientate the physician toward the diagnosis, limiting unnecessary and expensive tests. In particular, the presence at the birth of mild or severe kyphoscoliosis, together with joint hypermobility and the absence of the lingual and lower lip frenulum, should suggest an EDS [9–12]. The presence of kyphoscoliosis should evoke the kyphosclotic type of EDS prompting the execution of urine analysis. In presence of a normal LH/HP ratio a rare form due to FKBP14 mutation should be considered and genetically ruled out. The major EDS VI clinical features include congenital muscle hypotonia, congenital or early-onset kyphoscoliosis and generalized joint hypermobility with multiple dislocations/subluxations. Minor clinical features include skin hyperextensibility, easily bruisable skin, rupture/aneurysm of a medium-sized artery, osteopenia/osteoporosis, blue sclerae, hernia (umbilical or inguinal), pectus deformity, marfanoid habitus, talipes equinovarus, and refractive errors (myopia, hypermetropia) [13]. Moreover, there are other gene-specific minor criteria to be considered. In PLOD1 gene mutation disease, they are: skin fragility (e.g. atrophic scarring, friable skin), scleral/ocular fragility/rupture, microcornea, and facial dysmorphology (e.g. low-set ears, epicanthal folds, down-slanting fissures, synophrys, high palate). In FKBP14 gene mutation disease, minor criteria are congenital sensorineural, conductive or mixed hearing impairment, follicular hyperkeratosis, muscle atrophy, bladder diverticula. (Table 1) [14]. The presence of the first two major criteria plus the third major criteria or plus three minor criteria, suggest the EDS type VI diagnosis. The role of clinical criteria is to guide the execution of molecular testing, due to the wide genetic heterogeneity and phenotypic variability of EDS subtypes and the clinical overlap between many of these. The importance of the exact molecular determination is found in the impact it has on the patient’s care and allows a correct management of the problem. Long term follow-up in these patients is mandatory due to the variety of complications they can experience.

**Table 1:** Major and minor clinical criteria in EDS (adapted from) [14]

Major criteria	Minor criteria	Gene- specific minor criteria
	• Skin hyperextensibility	**PLOD1**
	• Easy bruisable skin	• Skin fragility
• Congenital muscle hypotonia	• Rupture/aneurysm of a medium-sized artery	• Scleral and ocular fragility/rupture
	• Osteopenia/osteoporosis	• Microcornea
• Congenital or early onset kyphoscoliosis	• Blue sclerae	**FKBP14**
	• Hernia (umbilical or inguinal)	• Congenital hearing impairment
• General Joint Hypermobolity	• Pectus deformity	• Follicular hyperkeratosis
	• Marfanoid habitus	• Muscle atrophy
	• Talipes equinovarus	• Bladder diverticula
	• Refractive error (myopia, hypermetropia)	

All patients should be aware of their skin fragility. The use of protective garments to be worn under clothes is recommended to reduce skin injuries, and expert surgeons should be involved in the need for suturing of any wounds. Since vascular ruptures have been reported, a cardiovascular assessment and regular follow up imaging may be required upon diagnosis. In particular, due to the risk of aortic root dilatation, it is advised to perform valve imaging by echocardiography (and possibly further examination with computed-tomography, angiography, magnetic resonance angiography) to measure the aortic root size by the age of 5 and to evaluate the presence of mitral or tricuspid valve prolapse [15]. Adults with severe kyphoscoliosis are at risk complications from chronic respiratory failure, recurrent pneumonia and cardiac failure. Recurrent joint dislocations are a common serious problem. Osteoporosis occurs in all individuals. The majority of patients have microcornea, but glaucoma and retinal detachment can also occur [13, 16].

## Data Availability

Not applicable.

## Abbreviations

EDS: Ehlers-Danlos syndrome
EDS VIA: kyphoscoliotic type EDS
SMA: Spinal Muscular Atrophy
PWS: Prader Willi syndrome
LH1: lysyl hydroxylase 1
LP: Lysyl Pyridoline
HP: Hydroxylysyl Pyridoline
CK: Creatin Kinase
MLPA: Multiple Ligation dependent Probe Amplification
